# A user-friendly platform for yeast two-hybrid library screening using next generation sequencing

**DOI:** 10.1371/journal.pone.0201270

**Published:** 2018-12-21

**Authors:** Marie-Laure Erffelinck, Bianca Ribeiro, Maria Perassolo, Laurens Pauwels, Jacob Pollier, Veronique Storme, Alain Goossens

**Affiliations:** 1 Ghent University, Department of Plant Biotechnology and Bioinformatics, Ghent, Belgium; 2 VIB Center for Plant Systems Biology, Ghent, Belgium; 3 Universidad de Buenos Aires, Facultad de Farmacia y Bioquímica, Departamento de Microbiología, Inmunología y Biotecnología, Cátedra de Biotecnología, Buenos Aires, Argentina; 4 CONICET-Universidad de Buenos Aires, Instituto de Nanobiotecnología (NANOBIOTEC), Buenos Aires, Argentina; University of Toronto, CANADA

## Abstract

Yeast two-hybrid (Y2H) is a well-established genetics-based system that uses yeast to selectively display binary protein-protein interactions (PPIs). To meet the current need to unravel complex PPI networks, several adaptations have been made to establish medium- to high-throughput Y2H screening platforms, with several having successfully incorporated the use of the next-generation sequencing (NGS) technology to increase the scale and sensitivity of the method. However, these have been to date mainly restricted to the use of fully annotated custom-made open reading frame (ORF) libraries and subject to complex downstream data processing. Here, a streamlined Y2H library screening strategy, based on integration of Y2H with NGS, called Y2H-seq, was developed, which allows efficient and reliable screening of Y2H cDNA libraries. To generate proof of concept, the method was applied to screen for interaction partners of two key components of the jasmonate signaling machinery in the model plant *Arabidopsis thaliana*, resulting in the identification of several previously reported as well as hitherto unknown interactors. Our Y2H-seq method offers a user-friendly, specific and sensitive screening method that allows identification of PPIs without prior knowledge of the organism’s ORFs, thereby extending the method to organisms of which the genome has not entirely been annotated yet. The quantitative NGS readout allows to increase genome coverage, thereby overcoming some of the bottlenecks of current Y2H technologies, which will further strengthen the value of the Y2H technology as a discovery platform.

## Introduction

Disentangling protein-protein interaction (PPI) networks is crucial for our understanding of cellular organization and function. To achieve this, a wide range of technologies to identify PPIs has been developed over the last decade [1, 2]. One of the most advanced and commonly used methods to identify PPIs *in vivo* under near-physiological conditions is affinity purification coupled to mass spectrometry (AP-MS) [3–5]. Equivalent comprehensive assays to specifically identify binary PPIs include protein domain microarrays and *in vivo* protein fragment complementation assays (PCAs) [6–10]. The principle of PCA is based on the fusion of two hypothetically interacting proteins (bait and prey) to two fragments of a reporter protein. Interaction between the bait and prey proteins results in the reassembly of the reporter protein, followed by its activation. The signal readout can be bioluminescence, fluorescence or cell survival. In the popular yeast two-hybrid (Y2H) method, the bait protein is fused to the DNA binding domain (DBD) and the prey (or prey library in the case of a comprehensive Y2H screening) is fused to the activation domain (AD) of a transcription factor (TF) [11]. Upon association of the hypothetical interactors, the TF is functionally reconstituted and drives the expression of a reporter gene that can be scored by selective growth. Typically, conventional medium-throughput Y2H library screenings are subject to laborious one-by-one clonal identification of interaction partners, but today, proteome-wide mapping of PPIs demands a high-throughput approach. This led for instance to the development of a matrix-based Y2H method that bypassed the inefficient identification by DNA sequencing [12]. Collections of bait and prey strains were automatically combined and arrayed on fixed matrix positions and PPIs were scored as visual readouts. A major drawback of this strategy is the need for pre-assembled libraries based on defined gene models and expensive robotics that are not accessible to every researcher.

Clonal identification of Y2H screening with DNA sequencing has a tremendous negative effect on the efficiency, cost and labor of the method. Furthermore, given the labor-penalty involved with increasing transformation titers, the clonal identification of Y2H interactions is usually not compatible with quantitative assessment of PPI abundances. Therefore, replacing the conventional Y2H screening strategy with a pool-based selection and global identification by NGS, can have three major implications: (i) cost reduction by high-capacity sequencing, (ii) higher sensitivity and (iii) quantification of the abundance of bait-specific interactions. The lab of Marc Vidal pioneered the implementation of the NGS technology for massive parallel Y2H screening in the Stitch-Seq method, mainly to map the human interactome. Herein, single amplicons, concatenating sequences of potentially interacting proteins, serve as template for NGS [13]. Nonetheless, this method remains laborious because it requires clonal isolation and several PCR rounds for PPI identification for each selected colony. The lab of Ulrich Stelzl developed the Y2H-seq method, thereby illustrating the advantage of NGS for Y2H towards scalability by mapping the protein methylation interactome [14]. In this strategy, the use of barcode indexing enables simultaneous sequencing of interacting preys of multiple separate baits in a single Illumina run. This strategy is based on mixing bait and prey pools prior mating, followed by selective growth, and deep-sequencing, but still requires a post-screen binary testing of interacting baits with each of the identified preys. The use of barcodes was further exploited in the Barcode Fusion Genetics‐Yeast Two‐Hybrid (BFG‐Y2H) method. This matrix-Y2H strategy uses Cre-recombinase to create intracellular chimeric barcodes that are derived from protein pairs, thereby enabling immediate identification and quantification of each interaction pair through NGS [15]. Prior to screening and NGS, isolation and sequencing of each barcoded bait and prey clone are essential to associate barcodes to ORFs, which may pose a cost restriction for massive screening purposes. Lewis et al., (2012) developed the Quantitative Interactor Screen Sequencing (QIS-Seq) approach, which provides a quantitative measurement of enrichment for each interactor relative to its frequency in the library without the use of barcode fusion proteins [16]. The latter was addressed in CrY2H-seq, which introduced a Cre-recombinase interaction reporter that endorses fusion of the coding sequences of two interacting proteins, followed by NGS to identify these interactions *en masse* [17]. The latter method was employed to uncover the transcription factor interactome of *A*. *thaliana*.

All of the above-mentioned Y2H-NGS strategies focus on increased capacity, efficiency and sensitivity, although they may face some lack in specificity, are laborious or do not fully exploit the quantification potential of NGS coupled to Y2H. Furthermore, construction of full-length ORF libraries is necessary, thereby restricting these methods to organisms of which the genomes are well annotated or to ‘defined’ gene models, which for instance cannot take alternative splicing, alternative start codon use or transcript processing into account.

Here, we discuss a user-friendly and standardized Y2H-NGS workflow (‘Y2H-seq’), complementary to the matrix-Y2H approaches, which allows rapid identification of interaction partners of a bait of interest in the organism of choice without the need for expensive robotics. The Y2H-seq screening method generates a quantitative readout that, through the use of control screens, allows to eliminate false-positive PPIs to boost the specificity of the method and thereby avoiding unnecessary downstream experimental binary interaction verification. Furthermore, the method is not dependent on predefined and prefabricated ORF libraries but on cDNA libraries, and is therefore principally applicable to every organism regardless of the annotation status of its genome. The functionality of our methodology is validated here by implementing it on two well-studied members of the jasmonate (JA) signaling cascade in the model plant *Arabidopsis thaliana*, i.e. TOPLESS (TPL) and Novel Interactor of JAZ (NINJA), respectively encoded by the loci AT1G15750 and AT4G28910 [18–26].

## Material and methods

### Gene cloning

All cloning was carried out by Gateway recombination (Thermo Fisher Scientific, Waltham, MA, USA). The full-length coding sequence of *IAA17*, *AT4G36480*, *AT1G34340*, *AT3G06850*, *AT4G05553*, *AT3G50000*, *AT3G54390*, *AT2G40260*, *AT3G05670*, *AT2G33550 and AT3G19860* were PCR-amplified (for primers, see S1 Table) using cDNA of Arabidopsis seedlings and recombined in the donor vector pDONR221. All the entry clones used in checkpoint 1 had previously been generated [18, 27].

### Binary Y2H analysis

Y2H analysis was performed as described [28] using the GAL4 system [28], in which bait and prey were fused to the GAL4-AD or GAL4-BD via cloning into pDEST22 or pDEST32, respectively. The *Saccharomyces cerevisiae* PJ69-4α yeast strain [29] was co-transformed with bait and prey constructs using the polyethylene glycol (PEG)/lithium acetate method. Transformants were selected on SD medium lacking Leu and Trp (Clontech, France). Three individual colonies were grown overnight in liquid cultures at 30°C and 10- or 100-fold dilutions were dropped on control (SD-Leu-Trp) and selective media (SD-Leu-Trp-His).

### Y2H screening

Yeast transformation was performed as described by Cuéllar-Pérez *et al*., (2013) [28]. The *S*. *cerevisiae* PJ69-4α yeast strain was transformed in two transformation rounds, respectively with 0.5 μg of bait plasmid DNA and 50 μg of cDNA prey library plasmid DNA using the PEG/lithium acetate method. At least 10^6^ transformants were plated on control (SD-Leu- Trp) and selective media lacking Leu, Trp and His supplemented with 5 mM 3-AT (Sigma-Aldrich, Saint Louis, MO, USA).

### Y2H cDNA library used to perform the Y2H screening

The ProQuest two-hybrid cDNA library was generated by cDNA synthesis from RNA extracted from *A*. *thaliana* suspension cells AT7, cloned into pEXP-AD502 vector (ProQuest), equivalent to pDEST22 vector (Thermo Fisher Scientific) and electroporated in the DH10B-Ton A (T1 and T5 phage resistance) cells (Thermo Fisher Scientific). The average insert size was 1.1 kb and the number of primary clones was 5.3 x 10^6^ cfu with a 100% insert coverage.

### Sanger sequencing

A minimum of ten random colonies of the Y2H screening plates were streaked out on solid SD-Leu-Trp-His selective medium with 5mM 3-AT (Sigma-Aldrich, Saint Louis, MO, USA) and incubated for 48 h at 30°C. Each streaked out colony was inoculated in liquid SD-Leu-Trp-His selective medium and incubated overnight at 30°C at 230 rpm. Subsequent yeast plasmid isolation was carried out using the Zymoprep Yeast Plasmid Miniprep I Kit (Zymo Research, Irvine, CA, USA) according to the manufacturer’s instructions. The cDNA inserts of the prey plasmids (pDEST22-insert) were PCR-amplified using backbone-specific primers (S1 Table) and Sanger-sequenced.

### Semi-quantitative qPCR

Colonies of the Y2H screening plates were dissolved and pooled in 10–15 mL of ultrapure water and plasmids were collected using the Zymoprep Yeast Plasmid Miniprep II kit (Zymo Research, Irvine, CA, USA). Prey constructs were amplified via PCR using Q5 High-Fidelity DNA Polymerase (New England Biolabs, Ipswich, MA, USA) and generic pDEST22 primers that bind to the GAL4AD and the region flanking the attR1 site (S1 Table). The following program was used: initial denaturation (98°C, 30 s), 35 amplification cycles (denaturation 98°C, 10 s; annealing 55°C, 30 s; elongation 72°C, 2.5 min), final extension (72°C, 5 min). The PCR mixture was purified using the CleanPCR kit (CleanNA, Alphen aan den Rijn, The Netherlands) and 40 ng of the purified PCR product was used for semi-quantitative qPCRs, which were carried out with a Lightcycler 480 (Roche Diagnostics, Brussels, Belgium) and the Lightcycler 480 SYBR Green I Master kit (Roche). Specific primers (S1 Table) and GoTaq DNA polymerase (Promega, Fitchburg, WI, USA) were used for amplification of 40 ng of purified PCR product in 3 technical replicates with the following program: initial denaturation (95°C, 5 min), 40 amplification cycles (denaturation 95°C, 30 s; annealing 60°C, 30 s; elongation 72°C, 60 s), final extension (72°C, 5 min). Three technical replicates per sample were performed. As a reference, a short sequence originating from the AD of pDEST22 was used. For the relative quantification with the reference gene, qBase was used [30].

### NGS data processing

The samples were sequenced by Illumina HiSeq 2000 125-bp paired-end reads. Data mapping and filtering were carried out through an in-house generated pipeline. To avoid sequencing artifacts such as read errors, primers, adapter and vector sequence contamination and PCR bias, a quality check was performed on the raw sequencing data. The quality control and trimming were performed with Trimmomatic [31]. Subsequently, the processed sequencing reads were mapped against the *Arabidopsis* reference genome, downloaded from TAIR (The Arabidopsis Information Resource, http://arabidopsis.org), by TopHat [32], which uses the Bowtie program as an alignment engine. In addition, TopHat requires SAM (Sequence Alignment/Map) tools to be installed. The cufflinks program was used to count the expression of each gene and report it as raw reads and FPKM. To determine possible interactors, following steps were taken. Genes with less than six read counts were not considered. Zero counts in the negative control sample were replaced by 1 to avoid division by 0. These genes were flagged to keep track of these imputations. FPKM values were calculated for each gene in both the sample and the negative control. Subsequently, the SNR was calculated for each gene as the ratio of the sample FPKM value to the negative control FPKM value. Genes with an SNR_NINJA/EMPTY_ or SNR_TPL-N/EMPTY_ higher than an arbitrary threshold calculated based on the 99.5^th^ percentiles of SNR_NINJA/EMPTY_ and SNR_TPL-N/EMPTY_, were considered to be potential interaction partners of the bait gene.

## Results

### Selection of baits

**Fig 1:**
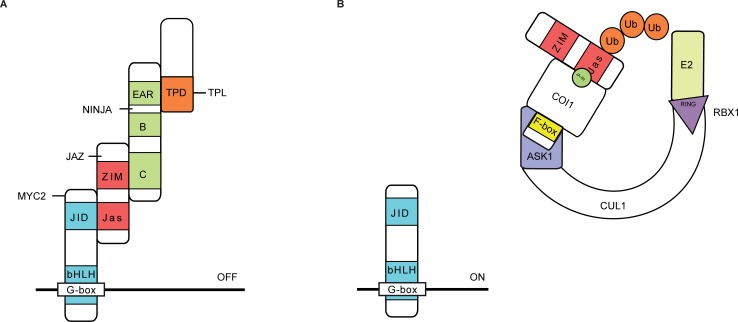
Function of TOPLESS and NINJA in JA signaling in *A*. *thaliana*. (A) In the absence of JAs, bHLH-type MYC TFs interact with the Jas domain of JAZ proteins that in turn interact with NINJA via their ZIM domain. The EAR motif of NINJA is essential for recruitment of the TPL co-repressors through the TPL domain (TPD). (B) In the presence of JA-Ile, JAZ proteins interact with the ubiquitin E3 ligase SCF^COI1^ complex, leading to the proteasomal degradation of JAZs and consequent release of the NINJA–TPL complex from the MYC TFs, which leads to the transcriptional activation of JA-responsive genes by de-repressed MYC TFs.

JAs are phytohormones that regulate the plant’s defense and modulate several developmental processes. The production of JAs via the oxylipin biosynthetic pathway leads to the accumulation of bioactive (+)-7-*iso*-jasmonoyl-L-isoleucine (JA-Ile). JA-Ile functions as a ligand between the F-box protein coronatine insensitive 1 (COI1) and the JA-ZIM (JAZ) repressor proteins, thereby promoting ubiquitination and subsequent proteasomal degradation of the JAZ proteins [33, 34]. Together with the TIFY8, peapod (PPD) and ZIM proteins, the JAZ proteins belong to the TIFY super-family [33, 35–38]. A key regulator in JA signaling in *A*. *thaliana* is the basic helix-loop-helix (bHLH) TF MYC2, encoded by the locus AT1G32640 [39, 40]. In the absence of JA-Ile, MYC2 can physically interact with the JAZ proteins via the Jas motif, which in turn recruit the transcriptional repressor TPL and TPL-related proteins (TRPs) through the adaptor protein NINJA [18]. NINJA acts as a transcriptional repressor that harbors an intrinsic TPL-binding ETHYLENE RESPONSE FACTOR (ERF)-associated amphiphilic repression (EAR) motif mediating its activity (Fig 1) [18]. NINJA can also interact with non-JAZ TIFY proteins, demonstrating its role in processes other than JA signaling [18, 35, 38, 41, 42]. Likewise, TPL is associated with various cellular processes through its capacity to interact with a compendium of diverse proteins [18–25, 38]. For instance, TPL can bind to PEAPOD proteins through the adapter proteins KIX8 and KIX9 to negatively regulate meristemoidal division in *A*. *thaliana* [38]. A role for TPL modulating brassinazole resistant 1 (BZR1)-regulated cell elongation and brassinosteroid-mediated control of shoot boundaries and root meristem development through interaction with the TF bri1-ems-suppressor 1 (BES1) has been described [23, 25]. TPL can also be recruited by CC-type glutaredoxins to target TGA-dependent promoters to control development- and stress-associated processes.

Because various direct interactors have been described for both NINJA and TPL proteins and because these are currently still heavily investigated for potential novel roles and links with different signaling pathways and cellular processes, NINJA and TPL were chosen as ideal bait proteins to develop, establish and validate our Y2H-seq methodology. Notably, whereas we used the full-length ORF of NINJA as a bait, for TPL only the amino-terminal region (AA 1–188; TPL-N) was used as a bait because this domain contains the lissencephaly homologous (LisH) dimerization and C-terminal to LisH (CTLH) motifs, which are together required and sufficient for interaction with transcriptional repressors through their EAR motif.

### The Y2H-seq flow-chart

**Fig 2:**
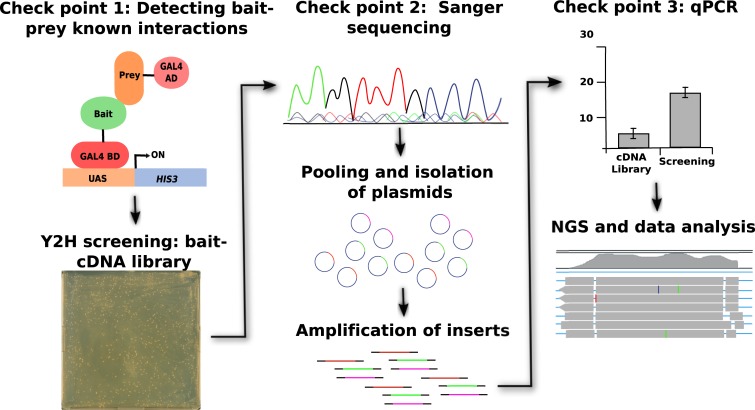
Y2H-seq workflow. The first checkpoint involves analysis of bait functionality through a Y2H assay with known interactors (preys). Alternatively, immunoblot analysis can be carried out in checkpoint 1 to validate protein expression of the fusion proteins in case no interactors are known of a particular bait prior to the screen. Subsequently, the actual Y2H screening with the functional bait is carried out by supertransformation of the bait yeast strain with the prey cDNA library. In checkpoint 2, the plasmids are extracted and Sanger sequenced for some of the colonies obtained on the selective plates. Next, pooling of all colonies on the plates is carried out, all plasmids from the pool are isolated in a single extraction and the inserts of the plasmids are amplified by PCR. In checkpoint 3, qPCR is performed to verify for enrichment of expected preys with a particular bait Y2H-seq PCR mix. For that, semi-quantitative qPCR analysis is carried out relative to the PCR product of a screen with an empty bait vector or the cDNA library itself. Finally, NGS and data analysis is performed to obtain a final list of putative interactors of the interested bait proteins.

An illustration of the general workflow of our Y2H-Seq strategy is given in Fig 2. As indicated above NINJA and TPL-N were used as baits and a Y2H cDNA library originating from *A*. *thaliana* AT7 suspension cells was used as prey.

After transformation of the Y2H reporter strain PJ69-4α with the bait plasmids, a first checkpoint is introduced, in which the bait strains were individually co-transformed with positive and negative control prey expression clones to verify functional expression of the baits, to exclude possible auto-activation and to corroborate binding with previously reported interaction partners (Fig 2). Next, the bait strains were used for Y2H-seq screening with the *A*. *thaliana* Y2H cDNA prey library. Simultaneously, a control screening was performed with the empty expression vector, which will hereafter be referred to as EMPTY.

Subsequent to five days of selective growth of the transformed yeast cells, the prey cDNA inserts of about ten individual yeast colonies per screen were Sanger-sequenced (Fig 2). This second checkpoint allowed us to confirm the retrieval of reported interactors as preys.

Subsequently, all yeast colonies that survived selective growth were pooled per screen and the cDNA inserts of the prey plasmid pools were amplified by PCR. A third checkpoint consisted of a qPCR analysis with specific primers for genes corresponding to known bait interactors, which allows to assess the representation of known interactors in both screens in a quantitative manner (Fig 2). Prey abundance was quantified relative to that in the *A*. *thaliana* Y2H cDNA library.

Upon complying the expectations of all three checkpoints, the amplicons of the pooled prey cDNA inserts were sequenced by NGS (Illumina HiSeq 2000 sequencing, 125-bp paired-end reads). The NGS-output was analyzed by an adapted RNA-Seq data processing pipeline, providing a quantitative selection of known and potentially new interactors of NINJA and TPL-N, using the EMPTY screen as control to eliminate false-positive interactions and to correct for the abundance of each prey represented by the Y2H cDNA library.

### Y2H-seq checkpoints

**Fig 3:**
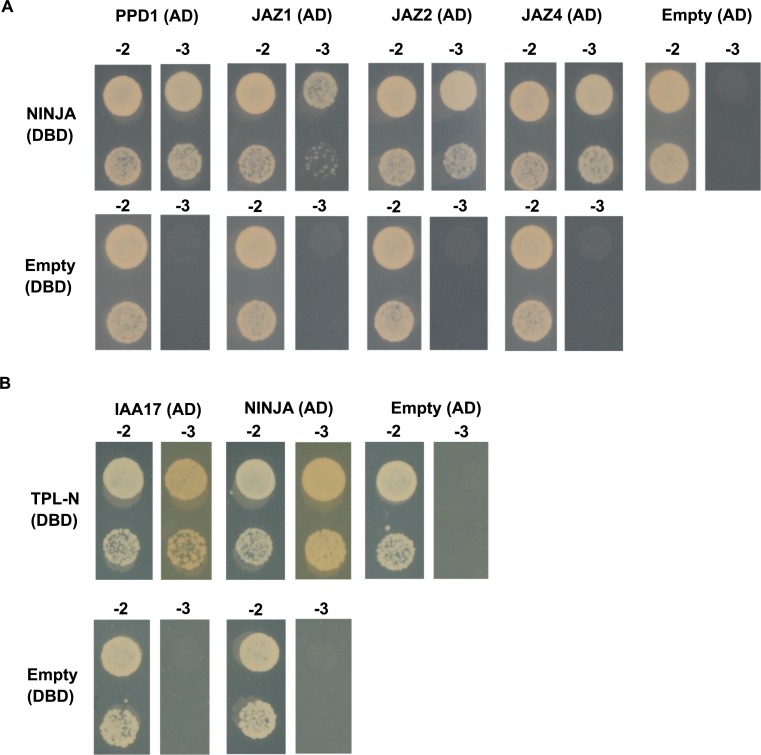
Y2H of the NINJA and TPL-N bait proteins with positive and negative control prey proteins. Y2H analysis of NINJA and TPL-N baits, fused to the DBD, and preys, fused to the AD, grown for 2 days on selective medium Synthetic Defined (SD)-Leu-Trp-His (-3). Transformed PJ69-4α yeast strains were also grown for 2 days on SD-Leu-Trp (-2) medium to confirm growth capacity. Direct interaction was confirmed between **(A)** NINJA and PPD1, JAZ1, JAZ2 and JAZ4, and **(B)** TPL-N and auxin/indole-3-acetic acid 17 (IAA17) and NINJA.

In a first checkpoint, we explore auto-activation and functionality of the bait strains. The bait strains were individually co-transformed with positive and negative control preys (Fig 3 and S2 Table) to determine the level of auto-activation of the bait strain and to check whether the bait protein is functionally expressed and consequently can bind previously reported interaction partners [18, 26, 38]. Alternatively, immunoblot analysis can be carried out to validate protein expression of the fusion proteins in case no interactors are known of a particular bait prior to the screen.

As expected, the binary interaction between the NINJA bait and the preys PPD1, JAZ1, JAZ2 and JAZ4 was confirmed (Fig 3A). Likewise, the TPL-N bait strain showed interaction with the preys auxin/indole-3-acetic acid 17 (IAA17) and NINJA (Fig 3B). Furthermore, neither of the bait strains exhibited auto-activation, which indicated that NINJA as well as TPL-N were functionally expressed in the bait strains.

**Fig 4:**
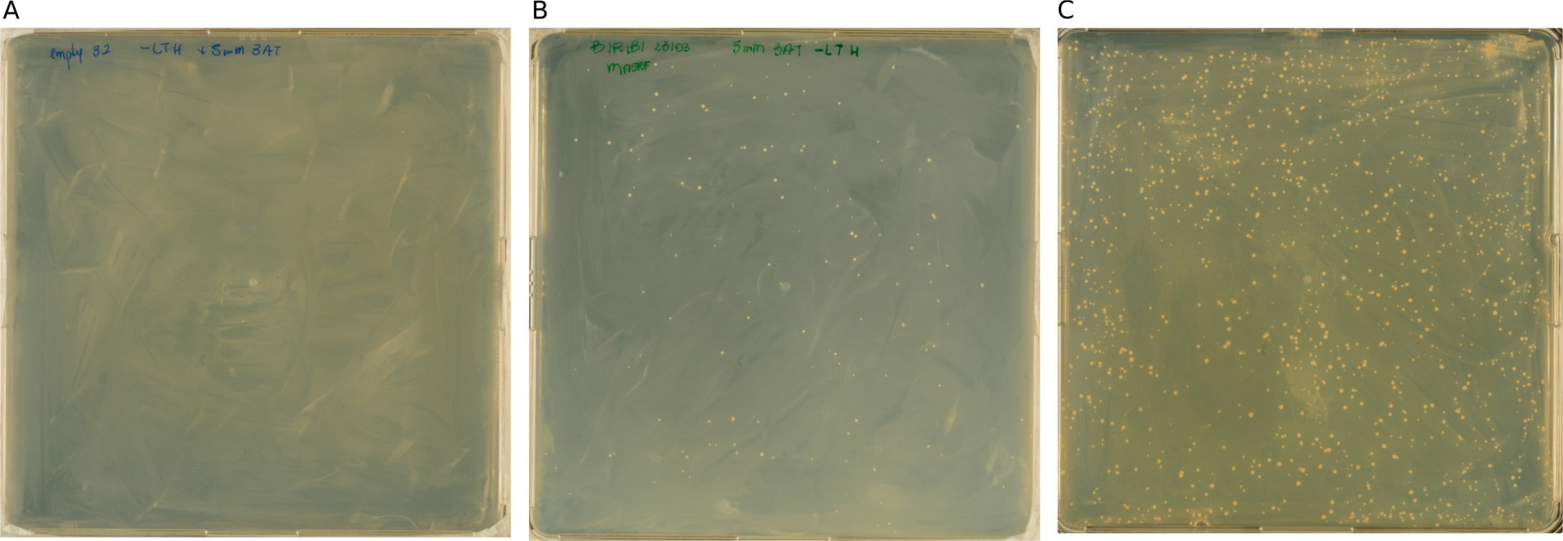
Y2H-seq selective growth. (A-C) The EMPTY (A), NINJA (B), and TPL-N (C) Y2H-seq screenings were performed on selective SD-Leu-Trp-His + 5mM 3-AT plates. Per bait, a single transformation reaction is carried out, after which each transformation mix is plated on 3 plates for selection. A representative plate for each bait is shown.

In a second checkpoint, we evaluate the functionality of the Y2H-seq screening with bait strains by Sanger sequencing. For the actual Y2H screening, the bait strains were transformed with the *A*. *thaliana* Y2H cDNA prey library, followed by transformation efficiency assessment and five days of selective growth (Fig 4 and S2 Table).

A minimum transformation efficiency of 1 x 10^6^ colony-forming units should be attained for a full Y2H cDNA library screening coverage [43]. This benchmark was achieved for all Y2H screenings we performed (Table 1 and S1 Fig).

**Table 1:** Transformation efficiency of Y2H screenings using EMPTY, TPL-N and NINJA as baits. To ensure a full screening coverage of the *A*. *thaliana* Y2H cDNA library, screening of at least 1 x 10^6^ yeast colonies is advised [43]. Titers are based on the numbers of colony forming units on control plates as illustrated for the TPL-N screen in S1 Fig.

**Bait**	**Transformation efficiency (# of colonies screened)**
**EMPTY**	1.23 x 10^7^
**NINJA**	3.85 x 10^6^
**TPL-N**	2.05 x 10^7^

A minimum of ten individual colonies per screening were isolated, plasmids purified and the cDNA inserts of the prey plasmids Sanger-sequenced. In this second checkpoint, several known interactors could already be identified (Table 2). The nine sequences originating from the NINJA screening corresponded to two unique interaction partners that were previously described as NINJA interactors [18]. Likewise, the 12 prey sequences that corresponded to potential interactors of TPL-N were derived from six different, all known interactors [19].

**Table 2:** Sanger sequencing of isolated NINJA and TPL-N preys.

**# Colonies**	**Gene description**		**Gene ID**
**NINJA**			
**8**	*A*. *thaliana* jasmonate-ZIM-domain protein 1 (JAZ1)	AT1G19180	
**1**	*A*. *thaliana* protein PEAPOD2 (PPD2)	AT4G14720	
**TPL-N**			
**4**	*A*. *thaliana* indole-3-acetic acid inducible 2 (IAA2)	AT3G23030	
**3**	*A*. *thaliana* indole-3-acetic acid inducible 28 (IAA28)	AT5G25890	
**2**	*A*. *thaliana* AGAMOUS-like 18 (AGL18)	AT3G57390	
**1**	*A*. *thaliana* indole-3-acetic acid inducible 4 (IAA4)	AT5G43700	
**1**	*A*. *thaliana* indole-3-acetic acid inducible 30 (IAA30)	AT3G62100	
**1**	*A*. *thaliana* indole-3-acetic acid inducible 9 (IAA9)	AT5G65670	

**Fig 5:**
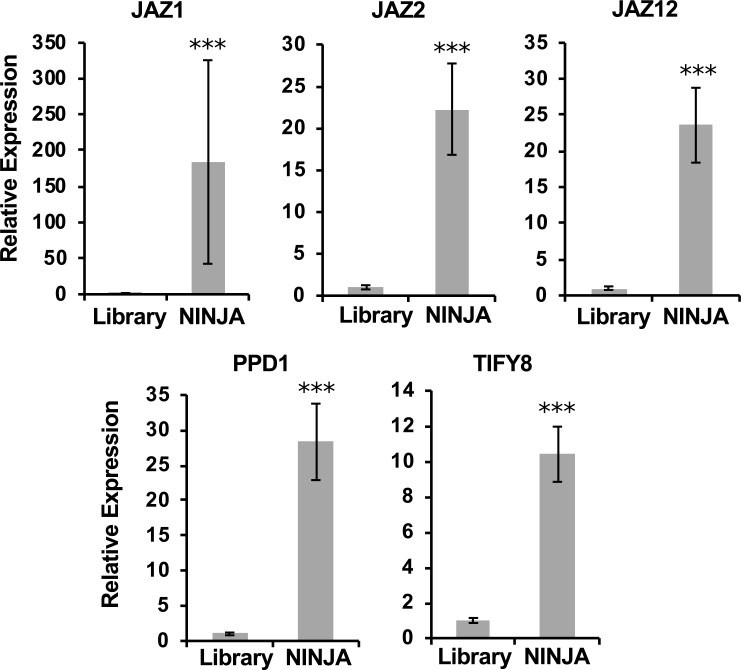
qPCR assessment of the NINJA Y2H-seq screen. *JAZ1*, *JAZ2*, *JAZ12*, *TIFY8* and *PPD1* were overrepresented in the PCR products of the NINJA screening compared to the PCR products of the *A*. *thaliana* cDNA library (Library). Statistical significance was determined by a Student’s t-test (***P<0.001).

In a third checkpoint, semi-quantitative qPCR is carried out, as a complementary approach to evaluate the quality of a Y2H-seq screening. In this third checkpoint, the quality of the Y2H-seq screening was further assessed. All selectively grown yeast colonies were pooled per screening (Fig 2) and cDNA inserts of the prey plasmid pools were PCR-amplified with vector-specific primers (S1 Table). To examine whether potential interaction partners of the baits were overrepresented relative to the cDNA library control, a qPCR was performed using prey-specific qPCR primers (S1 Table). In the NINJA screen, compared to the control library, the genes encoding JAZ1, JAZ2, JAZ12, TIFY8 and PPD1 were overrepresented (Fig 5), in agreement with previous literature reports [18, 35]. Hence, this shows the value of this qPCR assay set-up as a final checkpoint before the actual Y2H-seq analysis, at least for baits with a limited set of known interactors.

**Fig 6:**
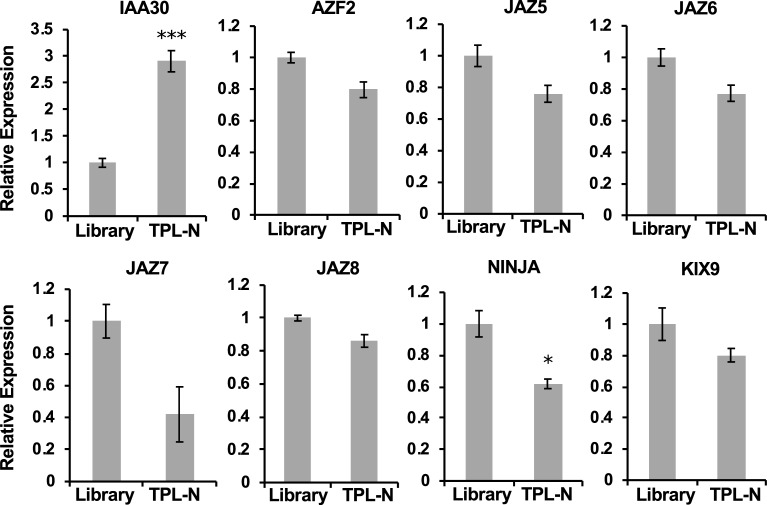
qPCR assessment of the TPL-N Y2H-seq screening. Only *IAA30* was overrepresented in the PCR products of the TPL-N screening compared to the *A*. *thaliana* cDNA library (Library) cDNA insert amplicons. Statistical significance was determined by a Student’s t-test (***P<0.001).

In contrast to NINJA, TPL can interact with potentially hundreds of proteins [19]. IAA30, the only protein identified in the checkpoint 2 that we also tested by qPCR, was enriched in the TPL-N pool. Conversely however, other EAR-motif containing proteins (from other families than the IAAs) that are known to interact with TPL, but were not readily identified in checkpoint 2, were not enriched (Fig 6, Table 2). Y2H cDNA library screenings are prone to false negatives, i.e. missing interactions, due among others to aberrant folding, clones with truncated genes or absence of the gene in the cDNA library. In the case of TPL-N, for example, the *NINJA* clone that is represented by the *A*. *thaliana* Y2H cDNA library was found to be truncated and missing the EAR domain necessary for binding with TPL-N.

### Beyond the checkpoints: NGS of the amplified prey cDNA inserts

The prey pool amplicons of the EMPTY, NINJA, and TPL-N screenings were used as input for NGS by Illumina HiSeq 2000 (125-bp paired-end reads). Here, we used a pipeline relying on TopHat for read mapping and Cufflinks for gene expression quantification. The method presented here aims to compare the NINJA and TPL-N Y2H-seq screens with the EMPTY control screen to enrich for specific interaction partners while maximally avoiding the retrieval of false-positive interactions.

First, a quality check was performed on the raw reads. Thereby, adapters, low-quality sequences and partial vector sequences were trimmed (EMPTY: 2,418,105 kept reads and 22,651 discarded; NINJA: 3,555,612 kept reads and 10,727 discarded; TPL-N: 2,908,652 kept reads and 33,094 discarded). Concomitantly, paired-end and orphan single-end reads were split. The processed reads were then mapped to the reference genome (TAIR10) using TopHat. To avoid overestimation of short genes, only one mate-pair per read was used for mapping. The resulting alignments were used as input for Cufflinks, which generates the raw expression quantification data for each of the analyzed raw sequencing files. For the subsequent analysis of the raw expression data, a Y2H-seq pipeline was drafted in R-studio.

Mapped genes in the TPL-N and NINJA Y2H screenings with raw read counts less than six were eliminated. Genes in the EMPTY screening that had no raw read counts were given an arbitrary value of 1 and flagged as imputed. After calculating the Fragments Per Kilobase of Exon Per Million Fragments Mapped (FPKM) values, the signal to noise ratio (SNR) was defined for NINJA and TPL-N compared to EMPTY. Intuitively, one would expect little NGS data to be derived from the EMPTY screening, given that no yeast cells survived selective growth (Fig 4). However, this was not the case and can be explained by the pooling method employed here: ‘scraping’ all yeast cells from the selection plates includes also dead or arrested yeast cells that may still contain intact prey plasmids. Hence, genes with a high representation in the cDNA library, and thus genes with a high expression level in *Arabidopsis* suspension cells, are identified in the EMPTY NGS data set.

Next, to allow setting relevant arbitrary thresholds, the 99.5^th^ percentiles of SNR_NINJA/EMPTY_ and SNR_TPL-N/EMPTY_ were calculated, leading to thresholds of 7.2 for NINJA and 6.0 for TPL-N screenings, respectively (Tables 3 and 4). With this first threshold, overall, from the 71 potential interactors of NINJA, seven were known to be interactors [18, 35], whereas for TPL-N, 12 out of the 51 potential interactors had been previously reported [26].

**Table 3:** Signal-to-noise ratio of the FPKM values of NINJA and EMPTY Y2H-seq screenings. Genes with SNR_NINJA/EMPTY_>7.2 were retained, listed and ranked from high to low SNR. Flagged genes are italicized. Previously reported interactors of NINJA are indicated in bold. Potential interactors that were tested for binary interaction in further validation assays are underlined.

	Gene ID	Gene-length	FPKM_EMPTY_	FPKM_NINJA_	SNR_FPKM_	Gene Alias	Full-length	TIFY domain
	**FPKM**_**NINJA**_**>100**							
1	**AT3G17860**	1588	0,731	1212,639	1659,075	JAI3/JAZ3/TIFY6B	Y	Y
2	**AT1G19180**	1329	27,074	20257,141	748,213	AtJAZ1/TIFY10A	Y	Y
3	**AT1G74950**	1280	4,081	2569,920	629,798	JAZ2/TIFY10B		Y
4	**AT5G13220**	1375	5,065	2735,323	540,063	JAS1/JAZ10/TIFY9	Y	Y
5	**AT4G14713**	1503	1,544	375,437	243,081	PPD1/TIFY4A	Y	Y
6	**AT1G17380**	1133	1,537	124,895	81,277	JAZ5/TIFY11A	Y	Y
7	**AT4G14720**	1568	3,701	294,079	79,455	PPD2/TIFY4B	Y	Y
	**FPKM**_**NINJA**_**<100**							
8	*AT4G36480*	2058	0,282	28,960	102,697	ATLCB1/EMB2779		
9	AT1G34340	1833	0,950	47,346	49,847			
10	AT3G06850	1730	1,342	33,645	25,074	BCE2/DIN3/LTA1		
11	AT4G05553	336	1,727	36,306	21,020		Y	
12	AT5G47810	1684	4,480	64,989	14,506	PFK2	Y	
13	AT3G03680	3308	0,175	2,213	12,612			
14	AT3G15760	843	0,688	8,269	12,011		Y	
16	AT3G02830	1695	0,342	4,113	12,011	PNT1/ZFN1	Y	
17	*AT2G34160*	636	0,912	10,412	11,411		Y	
18	AT4G17080	2053	0,283	3,056	10,810			
19	*AT3G06550*	2145	0,271	2,762	10,210	RWA2		
20	AT1G58150	276	2,103	20,205	9,609		Y	
21	AT1G29890	1901	0,916	8,801	9,609	RWA4		
22	AT2G33820	936	0,620	5,958	9,609	ATMBAC1	Y	
23	*AT1G73340*	1711	0,339	3,259	9,609		Y	
24	AT5G67440	2662	0,218	2,095	9,609	MEL2/NPY3		
25	AT1G32440	1908	0,304	2,740	9,009	PKp3		
26	*AT3G49350*	2220	0,261	2,355	9,009			
27	AT2G44830	2399	0,242	2,179	9,009			
28	AT5G27390	1040	1,116	9,719	8,708		Y	
29	AT2G30105	1377	0,421	3,544	8,408			
30	AT3G61790	1407	0,412	3,468	8,408			
31	AT4G39140	1600	0,363	3,050	8,408			
32	AT4G37880	1608	0,361	3,035	8,408			
33	AT1G26260	1622	0,358	3,008	8,408	CIB5		
34	AT4G02100	2085	0,278	2,340	8,408			
35	AT5G16000	2323	0,250	2,101	8,408	AtNIK1		
36	*AT2G23450*	2387	0,243	2,044	8,408			
37	AT3G42660	2862	0,203	1,705	8,408			
38	*AT1G13370*	648	0,896	6,992	7,807		Y	
39	AT1G66670	1196	0,485	3,788	7,807	CLPP3/NCLPP3	Y	
40	AT2G32340	1256	0,462	3,607	7,807		Y	
41	AT1G10660	1648	0,352	2,749	7,807			
42	AT3G16090	2120	0,274	2,137	7,807	AtHrd1A		
43	AT4G12120	2404	0,241	1,885	7,807	ATSEC1B		
44	AT3G23660	2568	0,226	1,764	7,807			
45	AT1G31440	1870	0,621	4,660	7,507			
46	AT3G44716	592	0,980	7,065	7,207		Y	
47	AT3G60640	643	0,903	6,505	7,207	ATG8G	Y	
48	AT2G34980	912	0,636	4,586	7,207	SETH1	Y	
49	*AT5G28330*	974	0,596	4,294	7,207		Y	
50	*AT4G25600*	1165	0,498	3,590	7,207			
51	AT2G18162	1231	0,471	3,398	7,207	CPuORF1	Y	
52	AT3G04730	1279	0,454	3,270	7,207	IAA16	Y	
53	AT4G26070	1342	0,432	3,117	7,207	ATMEK1/MKK1N	Y	
54	AT1G03687	1428	0,406	2,929	7,207			
55	AT1G06910	1528	0,380	2,737	7,207	TRFL7		
56	*AT1G52630*	1537	0,378	2,721	7,207		Y	
57	AT1G18570	1654	0,351	2,529	7,207	AtMYB51/BW51A	
58	*AT2G33580*	2239	0,259	1,868	7,207	LYK5		
59	AT5G04550	2580	0,225	1,621	7,207			
60	*AT4G03560*	2674	0,217	1,564	7,207	ATCCH1/ATTPC1		
70	AT4G02020	2876	0,202	1,454	7,207	EZA1/SDG10		
71	AT3G13690	3321	0,175	1,259	7,207			

**Table 4:** Signal–to-noise ratio of the FPKM values of TPL-N and EMPTY Y2H-seq screenings. Genes with SNR_TPL-N/EMPTY_>6 were retained, listed and ranked from high to low SNR. Flagged genes are italicized. Previously reported interactors of TPL are indicated in bold. Potential interactors that were tested for binary interaction in further validation assays are underlined. A ‘Y’ in bold font indicates the presence of an EAR domain in the wrong frame or in an untranslated region of the gene.

	Gene ID	Gene- length	FPKM_EMPTY_	FPKM_N-TPL_	SNR_FPKM_	Gene Alias	Full- length	EAR domain
	**FPKM**_**N-TPL**_**>100**							
1	**AT5G25890**	873	11,301	3997,602	353,736	IAA28/IAR2	Y	Y
2	**AT3G23030**	941	49,339	8581,581	173,933	IAA2	Y	Y
3	**AT5G43700**	1168	0,994	111,604	112,307	ATAUX2-11/IAA4	Y	Y
4	**AT4G28640**	1202	20,761	1121,873	54,037	IAA11	Y	Y
5	**AT4G29080**	1337	4,341	219,498	50,568	IAA27/PAP2	Y	Y
6	**AT3G15540**	970	7,180	266,613	37,135	IAA19/MSG2	Y	Y
7	**AT1G04250**	1087	249,863	8692,277	34,788	AXR3/IAA17	Y	Y
8	AT3G50000	1467	19,384	543,833	28,055	ATCKA2	Y	
9	**AT2G33310**	1820	64,093	1054,613	16,454	IAA13	Y	Y
10	AT2G46990	655	9,746	158,039	16,215	IAA20	Y	Y
11	**AT5G13790**	962	15,685	198,181	12,635	AGL15	Y	Y
	**FPKM**_**N-TPL**_**<100**							
12	AT3G54390	1341	3,0294	69,6555	22,9933		Y	
13	***AT4G37940***	715	0,8117	15,1114	18,6177	AGL21	Y	
14	*AT2G40260*	1233	0,4707	8,1976	17,4165		Y	
16	*AT3G58820*	1391	0,4172	5,2619	12,6120			**Y**
17	**AT1G51950**	1539	4,9022	56,1646	11,4570	IAA18	Y	Y
18	**AT1G04100**	1254	1,3884	15,8426	11,4108	IAA10	Y	Y
19	AT3G05670	3090	0,9391	9,2492	9,8493			Y
20	AT4G31620	1809	0,9624	9,2481	9,6091			**Y**
21	AT2G33550	1194	8,2629	77,0636	9,3265			Y
22	AT1G08290	1760	0,3297	2,9705	9,0085	WIP3		
23	AT3G19860	1288	3,1540	25,7074	8,1506	bHLH121	Y	
24	AT5G25160	959	1,2103	9,4494	7,8074	ZFP3	Y	Y
25	AT5G47110	1088	1,0668	8,3290	7,8074	LIL3:2		
26	AT3G04730	1279	0,4537	3,5426	7,8074	IAA16		Y
27	AT5G04550	2580	0,2249	1,7562	7,8074			
28	AT3G15760	843	0,6884	4,9614	7,2068		Y	
29	AT1G12270	1949	0,5955	4,2919	7,2068	Hop1		
30	AT3G47980	1097	0,5290	3,8126	7,2068			**Y**
31	AT1G02650	1542	0,3764	2,7124	7,2068			
32	AT2G38950	2482	0,2338	1,6851	7,2068			
33	AT3G19070	1041	1,6725	11,7184	7,0066			**Y**
34	AT1G28300	1317	0,8813	6,0868	6,9066	AtLEC2		**Y**
35	AT3G56250	669	0,8675	5,7308	6,6063		Y	
36	*AT1G01030*	1905	0,3046	2,0126	6,6063	NGA3		**Y**
37	AT1G61900	1913	0,3034	2,0041	6,6063			**Y**
38	AT1G79950	3123	0,1858	1,2276	6,6063			
39	AT3G43575	4332	0,1340	0,8850	6,6063			
40	AT5G36870	5616	0,2067	1,3033	6,3060	ATGSL09/atgsl9		
41	AT2G30540	680	4,2672	26,6529	6,2459			
42	AT2G38110	1845	4,0891	25,5028	6,2367	ATGPAT6		**Y**
43	AT2G25180	1980	3,2241	19,8913	6,1695	ARR12/AtARR12		**Y**
44	*AT1G53030*	530	1,0950	6,5762	6,0057			
45	*AT1G13680*	1180	0,4918	2,9537	6,0057			
46	*AT4G19540*	1215	0,4776	2,8686	6,0057	INDH/INDL	Y	
47	AT3G56160	1600	0,3627	2,1784	6,0057			
48	*AT5G03570*	1673	0,3469	2,0833	6,0057	ATIREG2/FPN2		
49	AT3G59150	1866	0,3110	1,8678	6,0057		Y	
50	*AT4G03560*	2674	0,2170	1,3034	6,0057	ATCCH1/ATTPC1/FOU2		
51	AT3G03680	3308	0,1754	1,0536	6,0057			

**Fig 7:**
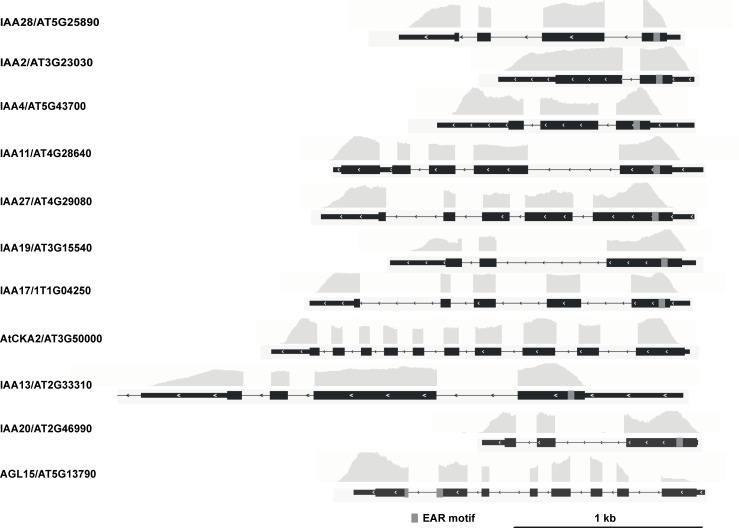
NGS coverage of the N-TPL interactors using cutoff of SNR_N-TPL/EMPTY_>6 and FPKM_N-TPL>_100. The depth of the NGS coverage for each gene, visualized by the coverage track, is aligned to the gene model. Coding sequences are represented by thick black boxes, 5’ and 3’ untranslated regions by thin black boxes and introns by thin black lines, respectively. The grey boxes in the gene model correspond to the EAR motif.

**Fig 8:**
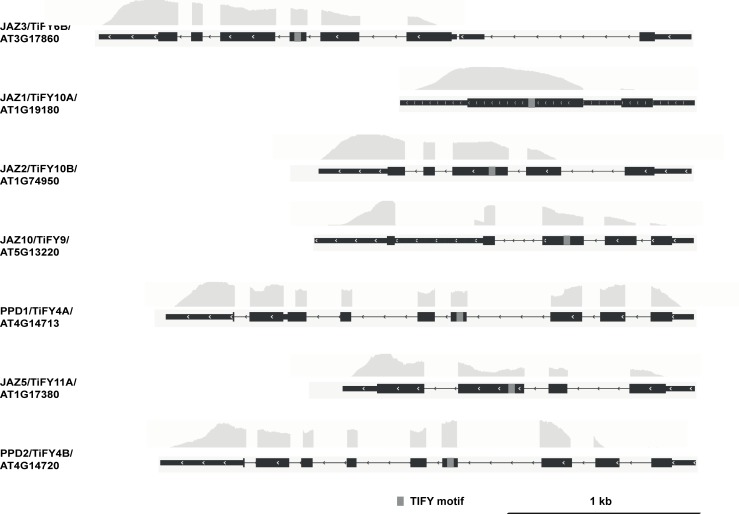
NGS coverage of the N-TPL interactors using cutoff of SNR_N-TPL/EMPTY_>6 and FPKM_N-TPL>_100. The depth of the NGS coverage for each gene, visualized by the coverage track, is aligned to the gene model. Coding sequences are represented by thick black boxes, 5’ and 3’ untranslated regions by thin black boxes and introns by thin black lines, respectively. The grey boxes in the gene model correspond to the EAR motif.

When super-implying a second threshold, in this case of >100 on the FPKM_NINJA_ and FPKM_TPL-N_ values, nearly all retained interactors were either reported already or very plausible. Indeed, in the case of NINJA, only TIFY-domain containing proteins were retained (Fig 7, Table 3). In the case of TPL-N, all but one of the retained proteins using this second threshold contained an EAR-motif [44], the conventional TPL recruitment domain (Fig 8, Table 4), and also includes proteins not yet individually reported as TPL-interactors, but belonging to multigene families such as the AGAMOUS-LIKE (AGL) and INDOLE-3-ACETIC ACID INDUCIBLE (IAA) proteins, many members of which have already been reported as TPL interactors [19, 26]. Together, this demonstrates the robustness and potential of the designed Y2H-seq platform.

**Fig 9:**
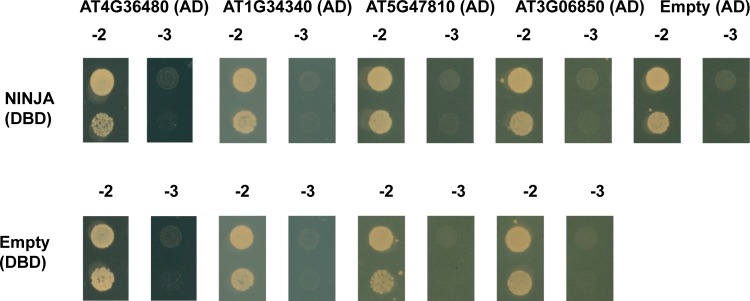
Y2H analysis of potential interaction partners of NINJA. Y2H analysis of NINJA, fused to the DBD, and potential interaction partners, fused to the AD of the GAL4 TF (in the pDEST22 vector), grown on selective medium SD-Leu-Trp-His (-3). Transformed PJ69-4α yeast strains were also grown on SD-Leu-Trp (-2) medium confirm growth capacity. No direct interactions could be observed for potential preys identified in the Y2H-seq with scoring values below the threshold of SNR_NINJA/EMPTY_>7.2 and FPKM_NINJA_ values>100.

To assess whether the retrieved preys that did not pass our stringent cut-offs, nonetheless represent true potential interactors of NINJA and N-TPL, additional Y2H experiments were carried out. For NINJA, the first four potential interaction partners with SNR_NINJA/EMPTY_>7.2 and FPKM_NINJA_<100 were tested in a binary Y2H assay (Table 3 and Fig 9). However, none of them showed interaction with NINJA, indicating that the installed threshold of SNR_NINJA/EMPTY_>7.2 and FPKM_NINJA_ >100 served as a good selection criterion, at least for NINJA.

**Fig 10:**
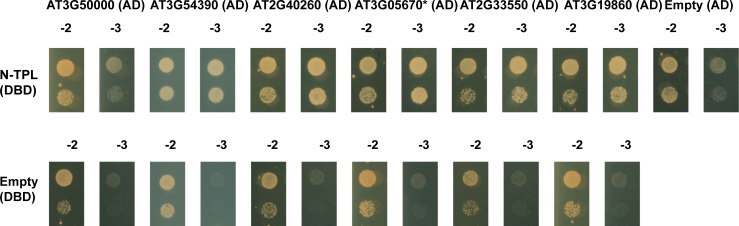
Binary Y2H validation of potential interaction partners of N-TPL. Y2H analysis of N-TPL, fused to the DBD, and potential interaction partners, fused to the AD of the GAL4 TF (in the pDEST22 vector), grown on selective medium SD-Leu-Trp-His (-3). Co-transformed PJ69-4α yeast strains were also grown on SD-Leu-Trp (-2) medium to confirm growth capacity. No direct interaction was confirmed between ATCKA2 encoded by AT3G5000 and N-TPL, in contrast to the interactions with all other potential interactors selected from the list with a threshold of SNR_NINJA/EMPTY_>7.2 and FPKM_NINJA_<100 values. * indicates a truncated version of the protein, as it was present in the Y2H cDNA library.

In the retained list of potential interactors using threshold SNR_TPL-N/EMPTY_>6 with FPKM_N-TPL_>100 values, the one candidate ATCKA2 (AT3G50000) that did not contain an EAR-domain was tested for direct interaction with N-TPL in a Y2H assay, besides five candidates with FPKM_N-TPL_<100 (Table 4 and Fig 10). For the latter set, we specifically avoided to pick candidates from the AGL and IAA families, which are most likely true, but less abundant interactors, and chose both candidates with and without an EAR domain. ATCKA2 interaction with N-TPL could not be confirmed with binary Y2H, suggesting it was a false positive caused by the Y2H-seq pipeline. In contrast however, interaction between TPL-N and the five other candidates were all confirmed, demonstrating that they do not represent artefacts of the Y2H-seq methodology and may be true interactors. Hence, in contrast to NINJA, this implicates that the arbitrary threshold of SNR_TPL-N/EMPTY_>6 with FPKM_N-TPL_>100 was too stringent for N-TPL. Perhaps this may be due to the pleiotropic function of TPL, which has an exceptionally high number of protein interactors, often from multigene families. For proteins such as NINJA, with a more defined role and a well-defined set of interactors, a stricter threshold may be justified. For proteins such as TPL, one may need to be more relaxed in determining candidate interactors. As exemplified here, this leads to the identification of potential novel interactors from gene families previously unreported to be capable of interacting with TPL, including EAR-domain containing proteins such as the RING/U-box protein AT3G05670, or proteins that do not contain an EAR domain such as the putative TF AT3G54390, the homeodomain TF AT2G40260 and the bHLH TF AT3G19860 (Table 4 and Fig 10).

## Discussion

Here, we present a newly designed Y2H-seq strategy to identify PPIs, which enables exploiting the full qualitative and quantitative potential of Y2H library screenings in an unprecedented way. Our method circumvents multiple shortcomings of a conventional Y2H library screening. As such, for instance consumable and DNA sequencing costs are significantly cut by using a pool-based NGS-strategy instead of the conventional isolation, manipulation and sequencing of individual yeast clones that survive the screening selection. Moreover, a higher PPI coverage can be achieved in our Y2H-seq strategy by increasing library titers. A factor that will determine the impact of future Y2H-seq screenings more than ever, will be the choice and the quality of the Y2H cDNA library. For instance, full-length protein libraries may mask PPIs by steric hindrance, hence the use of more complex Y2H cDNA libraries encoding protein fragments as well as full-length proteins may now be considered, and screened in one effort, which could lead to a more comprehensive coverage of the PPI space. The utility of fragment-based Y2H approaches has previously been demonstrated [45, 46]. By playing with sample preparations to generate cDNA libraries, one could increase the genome coverage with no extra effort in the Y2H screening. For instance, different organs from a single plant, different developmental stages of a single organ, or explants subjected to different environmental cues or chemicals can now be pooled in a single cDNA library. This will allow expanding the number of genes screened in a single event, as well as different versions of the same gene, e.g. following expression after alternative splicing or translation start events. As such, the Y2H-seq strategy can provide a more effective way to map the PPI potential of a bait protein, allowing further exploration of biological pathways and their regulation. Furthermore, the use of cDNA libraries makes it possible to identify novel interaction partners of organisms of which the genome has not been fully annotated yet, unlike the use of ORF libraries based on known and completely fixed gene models.

The Y2H-seq strategy implements a quantitative readout system, with a straightforward and adaptable scoring procedure. The comparison of quantitative NGS readouts from a Y2H-seq screen with a particular bait protein to those from a control screen with the ‘empty’ control vectors is essential to discriminate potential true interactors from background from the library, for instance from abundantly expressed genes. Likewise, exhaustive screening of a particular cDNA library with different baits may allow comparison of the readouts of the different screens to further discriminate specific interactors screenings from ‘sticky’ proteins and thereby further increase the efficiency of the method. Indeed, as is also the case with other PPI discovery methods, such as tandem affinity purification [47, 48], a specific ‘blacklist’ of returning Y2H-seq interactors for each cDNA library can be composed by marking common interactors of seemingly unrelated bait proteins. This may allow fine-tuning the thresholds to be set up in the filtering of the Y2H-seq NGS data, and thereby enable determining robust priority lists and reducing laborious and needless downstream validation assays to a minimum.

Finally, this strategy can also easily be extended to Y1H screenings, for which the same cDNA library could be screened, but for which considerably higher false-positive rates are typically obtained as compared to Y2H screenings [49, 50]. As such, we anticipate that the cost and labor reduction along with the increased detection and quantification potential of our Y2H-seq strategy can give an important upgrade to this long-existing, but far from fully exploited screening tool.

## Supporting information

S1 Fig Control plates used to determine the number of colony-forming units to calculate the efficiency of transformation and library titer for the TPL-N screening. (DOCX)

S1 Table Primers used in this study. (DOCX)

S2 Table Yeast strains generated in this study. (DOCX)
