# Supplementary material for: A user-friendly platform for yeast two-hybrid library screening using next generation sequencing
Source: PLoS One. 2018 Dec 21;13(12):e0201270. doi: 10.1371/journal.pone.0201270 (PMC6303091; doi:10.1371/journal.pone.0201270)
Supplement: S1 Fig — (DOCX) [file pone.0201270.s001.docx]

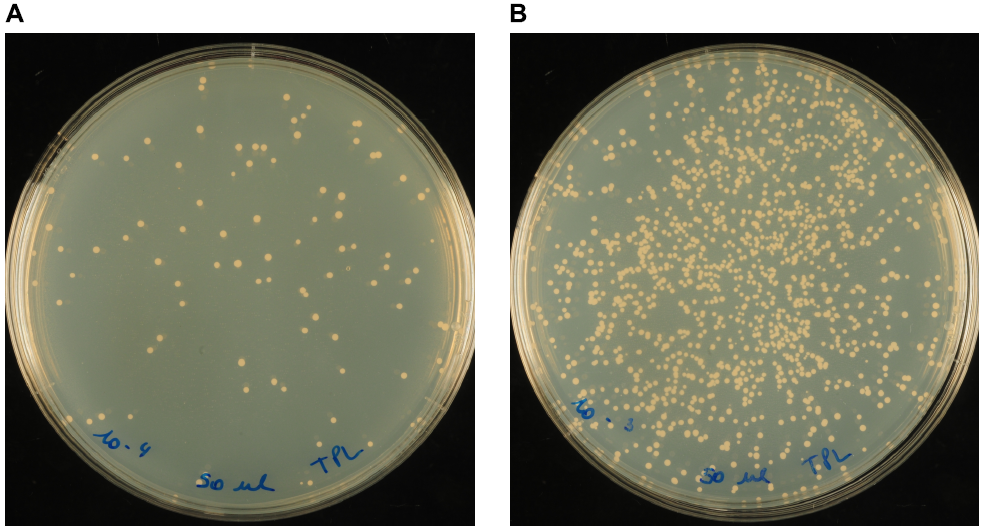


**S1 Fig. Control plates used to determine the number of colony-forming units to calculate the efficiency of transformation and library titer for the TPL-N screening.** (A-B) 10,000 (A) and 1,000-fold (B) dilutions of the transformed yeasts were plated out on medium lacking the Leu and Trp for selection for bait and prey plasmids, respectively.
