# Supplementary material for: A user-friendly platform for yeast two-hybrid library screening using next generation sequencing
Source: PLoS One. 2018 Dec 21;13(12):e0201270. doi: 10.1371/journal.pone.0201270 (PMC6303091; doi:10.1371/journal.pone.0201270)
Supplement: S1 Table — (DOCX) [file pone.0201270.s002.docx]

**S1 Table. Primers used in this study.**

| Description | | Sequence |
| --- | --- | --- |
| IAA17 cloning primer Fw | 5’- AAAAAGCAGGCTCCATGATGGGCAGTGTCGAGCT-3’ | |
| IAA17 cloning primer Rv | 5´-AGAAAGCTGGGTCTCMAGCTCTGCTCTTGCACTTCT-3’ | |
| AT4G36480 cloning primer Fw | 5’- GGGGACAAGTTTGTACAAAAAAGCAGGCTCCATGGCTTCGAATCTCGTGGAAAT-3’ | |
| AT4G36480 cloning primer Rv | 5’- GGGGACCACTTTGTACAAGAAAGCTGGGTCTCAGGACTTGAGTAGAAGCT-3’ | |
| AT1G34340 cloning primer Fw | 5’- GGGGACAAGTTTGTACAAAAAAGCAGGCTCCATGGAAGAAGAAGATCTGAT-3’ | |
| AT1G34340 cloning primer Rv | 5’- GGGGACCACTTTGTACAAGAAAGCTGGGTCTCAGGATGTGGATGTTGTT-3’ | |
| AT3G06850 cloning primer Fw | 5’- GGGGACAAGTTTGTACAAAAAAGCAGGCTCCATGATCGCGCGACGGATCTGGCG-3’ | |
| AT3G06850 cloning primer Rv | 5’- GGGGACCACTTTGTACAAGAAAGCTGGGTCTCATCTCATTTGAAGCATCAGCAGCTCC-3’ | |
| AT4G05553 cloning primer Fw | 5’- GGGGACAAGTTTGTACAAAAAAGCAGGCTCCATGTGTGGTGTAGAAGAAAGT-3’ | |
| AT4G05553 cloning primer Rv | 5’- GGGGACCACTTTGTACAAGAAAGCTGGGTCTTACTCCGTACAAGTGCTCA-3’ | |
| AT3G50000 cloning primer Fw | 5’- GGGGACAAGTTTGTACAAAAAAGCAGGCTCCATGCACCTAATCTTCTTCTTCTCC-3’ | |
| AT3G50000 cloning primer Rv | 5’- GGGGACCACTTTGTACAAGAAAGCTGGGTCTCATTGAGTCCTCATTCTGCTGC-3’ | |
| AT3G54390 cloning primer Fw | 5’- GGGGACAAGTTTGTACAAAAAAGCAGGCTCCATGGAGAACGGAGAATCTAA-3’ | |
| AT3G54390 cloning primer Rv | 5’- GGGGACCACTTTGTACAAGAAAGCTGGGTCTCAATTTCTTCCGATCCTTAATGAAGA-3’ | |
| AT2G40260 cloning primer Fw | 5’- GGGGACAAGTTTGTACAAAAAAGCAGGCTCCATGAGATCAAGCAGCCAAAATTCTG-3’ | |
| AT2G40260 cloning primer Rv | 5’- GGGGACCACTTTGTACAAGAAAGCTGGGTCTCACAGAGTGAGATCAAGGGGACTTGCC-3’ | |
| AT3G05670 cloning primer Fw | 5’- GGGGACAAGTTTGTACAAAAAAGCAGGCTCCATGGGAAAGAGAAACACGTCTTGG-3’ | |
| AT3G05670 cloning primer Rv | 5’- GGGGACCACTTTGTACAAGAAAGCTGGGTCTTAGTGGAGCCCTAGACTC-3’ | |
| AT2G33550 cloning primer Fw | 5’- GGGGACAAGTTTGTACAAAAAAGCAGGCTCCATGGCTCTGGAACAGTTAGGATTAG-3’ | |
| AT2G33550 cloning primer Rv | 5’- GGGGACCACTTTGTACAAGAAAGCTGGGTCTCACATCTTATCCGCGATTTTTGCCACAGC-3’ | |
| AT3G19860 cloning primer Fw | 5’- GGGGACAAGTTTGTACAAAAAAGCAGGCTCCATGGGGATAAGAGAAAATGGAATAATGCTTG-3’ | |
| AT3G19860 cloning primer Rv | 5’- GGGGACCACTTTGTACAAGAAAGCTGGGTCTCATTTTGCATCATCAGGTTTTTGGCCACCAGC-3’ | |
| JAZ1 (AT1G19180) qPCR primer Fw | | 5’- TTCTGAGTTCGTCGGTAGCC -3’ |
| JAZ1 (AT1G19180) qPCR primer Rv | | 5’- CACGTCTGTGAGAAGCTAGGC -3’ |
| JAZ2 (AT1G74950) qPCR primer Fw | | 5’- CTCTTTAGCCTGCGAACTCC -3’ |
| JAZ2 (AT1G74950) qPCR primer Rv | | 5’- TTGGTATGGTGCCTTTGATG -3’ |
| JAZ5 (AT1G17380) qPCR primer Fw | | 5’- AAAGATGTTGCTGACCTCAGTG -3’ |
| JAZ5 (AT1G17380) qPCR primer Rv | | 5’- CCCTCCGAAGAATATGGTCA -3’ |
| JAZ6 (AT1G72450) qPCR primer Fw | | 5’- TTCATCGATTCTTTGCTAAACG -3’ |
| JAZ6 (AT1G72450) qPCR primer Rv | | 5’- ATCGATGGAGCAACCATCTC -3’ |
| JAZ7 (AT2G34600) qPCR primer Fw | | 5’-ATGCGACTTGGAACTTCGCCTT-3’ |
| JAZ7 (AT2G34600) qPCR primer Rv | | 5’-AGAGCTGCTTGATTCGTCCAACG-3’ |
| JAZ8 (AT1G30135) qPCR primer Fw | | 5’-CGATCGCAAGCAGAGAAATG-3’ |
| JAZ8 (AT1G30135) qPCR primer Rv | | 5’-GATCCGACCCGTTTGAGGAT-3’ |
| JAZ12 (AT5G20900) qPCR primer Fw | | 5’- CATCTAATGTGGCATCACCAG -3’ |
| JAZ12 (AT5G20900) qPCR primer Rv | | 5’- TGCCTCCTTGCAATAGGTAGA -3’ |
| NINJA (AT4G28910) qPCR primer Fw | | 5’-AAGTGATTCGGGTCAACAGC-3’ |
| NINJA (AT4G28910) qPCR primer Rv | | 5’-GGTTGGAAGAAGAACCACCA-3’ |
| PEAPOD1 (AT4G14713) qPCR primer Fw | | 5’-AAAGATGGCCACAAGACGAC-3’ |
| PEAPOD1 (AT4G14713) qPCR primer Rv | | 5’-GGACACTTTTTGGCCTTTGA-3’ |
| AZF2 (AT3G19580) qPCR primer Fw | | 5’-ATTCAACAGCTCCGACCATC-3’ |
| AZF2 (AT3G19580) qPCR primer Rv | | 5’-GGCTCCTTTCTTCCGATACC-3’ |
| KIX9 (AT4G32295) qPCR primer Fw | | 5’-ATCATGTATTCCAAAGCCAATTC-3’ |
| KIX9 (AT4G32295) qPCR primer Rv | | 5’-CGGTCTAAAAGGGTCTTCATGT-3’ |
| TIFY8 (AT4G32570) qPCR primer Fw | | 5’-CGTCTCCGACAGACAGAACA-3’ |
| TIFY8 (AT4G32570) qPCR primer Rv | | 5’-CCTGAAAACCGATTGCTCAT-3’ |
| IAA30 (AT3G62100) qPCR primer Fw | | 5’-TTCAATGCTTCAATCCTTTGG-3’ |
| IAA30 (AT3G62100) qPCR primer Rv | | 5’-AGCACGTGACTCTTCTCACTACA-3’ |
| GAL4AD pDEST22 Rv | | 5’-GGTTTGGTGGGGTATCTTCA-3’ |
| pDEST22 Fw Sanger sequencing | | 5’-TATAACGCGTTTGGAATCACT-3’ |
| pDEST22 Rv Sanger sequencing | | 5’-AGCCGACAACCTTGATTGGAGAC-3’ |
