# Supplementary material for: A user-friendly platform for yeast two-hybrid library screening using next generation sequencing
Source: PLoS One. 2018 Dec 21;13(12):e0201270. doi: 10.1371/journal.pone.0201270 (PMC6303091; doi:10.1371/journal.pone.0201270)
Supplement: S2 Table — (DOCX) [file pone.0201270.s003.docx]

**S2 Table. Yeast strains generated in this study.**

| Strains generated for binary Y2H assays |
| --- |
| PJ69-4α; pDEST32[TPL-N];pDEST22[IAA17] |
| PJ69-4α; pDEST32[TPL-N];pDEST22[NINJA] |
| PJ69-4α; pDEST32[TPL-N];pDEST22[EMPTY] |
| PJ69-4α; pDEST32[NINJA];pDEST22[PPD1] |
| PJ69-4α; pDEST32[NINJA];pDEST22[JAZ1] |
| PJ69-4α; pDEST32[NINJA];pDEST22[JAZ2] |
| PJ69-4α; pDEST32[NINJA];pDEST22[JAZ4] |
| PJ69-4α; pDEST32[NINJA];pDEST22[EMPTY] |
| Strains generated for Y2H-seq screening |
| PJ69-4α; pDEST32[TPL-N] |
| PJ69-4α; pDEST32[NINJA] |
| PJ69-4α; pDEST32[EMPTY] |
